# Assessment of Efficacy on the Treatment of Localized Vitiligo With a Combination of 308 nm Excimer Light and 2940 Erbium Laser: A Retrospective Study

**DOI:** 10.1111/jocd.16715

**Published:** 2024-12-08

**Authors:** Yihui Xie, Weimin Shi, Wenhao Yin, Xingyu Mei

**Affiliations:** ^1^ Department of Dermatology, The Affiliated Hospital of Jiaxing University The First Hospital of Jiaxing Jiaxing Zhejiang China; ^2^ Department of Dermatology, Shanghai General Hospital Shanghai Jiao Tong University School of Medicine Shanghai China

**Keywords:** 2940 erbium laser, 308 nm excimer light, vitiligo

## Abstract

**Background:**

A 308 nm excimer light is an effective treatment for vitiligo, with fewer treatment times, rapid obvious effects, relatively complete pigment recovery, good patient compliance, and safe and effective treatment for children. The disadvantage of this treatment is that some patients have a plateau during which the skin lesion reduction rate slows down or stalls after multiple treatments.

**Methods:**

Treatment data were collected from all vitiligo patients in the Dermatology Department, distinguishing between those who underwent 308 nm excimer light treatment alone and those who received a combination of 308 nm excimer light and 2940 erbium laser treatment.

**Results:**

Of the 104 patients, 60 were treated with 308 nm excimer light only, and 44 were treated with a combination of a 2940 erbium laser and 308 nm excimer light. In contrast to the treatment of 308 nm excimer light alone, the combined treatment of the 2940 erbium laser and 308 nm excimer light significantly increased the speed and degree of repigmentation in vitiligo.

**Conclusion:**

Our study demonstrates that the combination of 308 nm excimer light and 2940 nm erbium laser treatment can improve the treatment response for vitiligo, decrease the occurrence of treatment plateaus associated with 308 nm excimer light, and improve efficacy.

**Trial Registration:**

ChiCTR2000036712 (24/08/2020)

## 1 Introduction

Vitiligo, a condition characterized by white patches on the skin, is an autoimmune disease [1, 2]. The localized or generalized depigmented lesions are a result of the reduction or absence of functional melanocytes in the skin and hair follicles [3]. Pathogenesis is unclear and is related to genetic and nongenetic factors [4, 5, 6]. Vitiligo is a prevalent condition that may affect various regions of the body and manifest at any age [7].

Therapeutic options, such as narrowband ultraviolet B (NB‐UVB) therapy and the 308 nm excimer light and 308 nm excimer laser, have been proven to be both safe and effective for treating vitiligo in both children and adults [8, 9]. NB‐UV light refers to UV light with a wavelength of 311 nm, a commonly used treatment method for vitiligo in recent years, and can stimulate the remaining melanocytes' proliferation and migration [10]. Both the 308 nm excimer light and 308 excimer laser demonstrate similar efficacy in treating vitiligo [11]. While their mechanisms of action are alike, they utilize distinct luminescence processes, and there is no notable difference in the rates of color restoration in the affected areas [11]. The 308 nm excimer light belongs to the ultraviolet category, which is an incoherent single‐frequency light source with 10 times the energy of NB‐UVB [12]. The 308 nm excimer laser uses xenon chloride gas as the irradiation source and emits a UV laser at 308 nm to induce T cell death, improve local immunity, and stimulate melanocytic hyperplasia [13, 14]. Compared with NB‐UVB, the 308 nm excimer light and 308 nm excimer laser require fewer treatments, faster obvious effect, more complete pigment recovery, good patient compliance, and are also safe and effective in children [15, 16]. The disadvantage is time‐consuming and difficult to treat generalized skin lesions [17, 18]. In contrast to the 308 nm excimer laser, the 308 nm excimer light offers several advantages, including a reduced power density, an extensive irradiation area, a lack of consumables, a brief irradiation duration, and lower treatment expenses.

Many experiments have shown that, compared with the current commonly used treatments, the 308 nm excimer light treatment for stable vitiligo has a faster effect, higher efficacy, and fewer side effects [19, 20]. However, previous research and clinical treatments have shown that there is a plateau in the 308 nm excimer light treatment. May the sequential therapy of 308 nm excimer light and 2940 erbium laser/CO_2_ fractional laser be more effective? [21, 22]

A 2940 erbium laser is an exfoliative fractional laser. Fractional laser produces multiple fractional arrangements of small light beams on the skin to form microthermal zones: Heat can make the tissue in the skin lesion contract; trauma stimulates the melanocytes' division and proliferation by promoting the secretion of cytokines and growth factors; and the disruption of local barrier function promotes the penetration of topical drugs and UV light and increases the therapeutic effect. The undamaged keratinocytes around them migrate rapidly, multiply and repair the microtherapeutic area, stimulate dermal collagen hyperplasia, and promote the reconstruction of dermal structure to achieve the purpose of treatment [23, 24].

Compared with erbium laser treatment, the CO_2_ fractional laser has more thermal damage, more pain, and longer healing time. The adverse reactions of CO_2_ fractional laser include pain, burning sensation, erythema, edema, isomorphic reaction, scar formation, etc. Due to its low thermal effect, the erbium laser has a lower depth of action on the skin than the CO_2_ laser and has fewer side effects after surgery; hence, the pain is very low. The advantages of an erbium laser include small pain, short recovery time, a good hemostatic effect, and the abililty to accurately control the thermal penetration of deep tissue and increase the absorption of external drugs [22]; in addition, it can stimulate keratinocytes to produce metalloproteinase [25] and promote the proliferation and migration of melanocytes, which is conducive to the treatment of vitiligo. Therefore, we believe that the 308 nm excimer light combined with the 2940 erbium laser is a better choice than the CO_2_ fractional laser.

We hope to explore the efficacy of the 308 nm excimer light together with the 2940 erbium laser in patients with vitiligo by tracing previous cases. In the future, we further optimize the treatment options of the patients.

## 2 Method

### 2.1 Patient Screening

This is a retrospective observational study. Patients with potential pregnancy or lactation, history of photosensitive disease, and any other conditions that could affect the therapy were excluded. Skin lesions were all monitored for 26 weeks. For 100% repigmentation skin lesions, the time of complete repigmentation was documented. Inclusion criteria: Individuals diagnosed with nonsegmental, undetermined vitiligo, classified as having type II or III skin, without the presence of leukotrichia. The area of each skin lesion was less than 1% of the overall body surface area [26]. During treatment, all patients received oral folic acid tablets, vitamin B1, and topical 0.1% tacrolimus ointment.

Groups: Group A: Combined with the 308 nm excimer light and 2940 erbium laser, with fortnightly treatments, each treatment was given 308 nm excimer light before 2940 erbium laser; Group B: 308 nm excimer light only, with fortnightly treatments [27].

Device information: Alma Lasers, Harmony XL, Israel. Base parameter settings: 308 nm excimer light: initial dose, 2000 mJ/cm^2^; pulse width, 40 msec; 2940 erbium laser: 1900 mJ/pixel and long pulse width mode. The energy was slightly adjusted according to the operator's experience and the patient's initial treatment response: If the erythema reaction time was < 1 week, the energy of 308 nm excimer light was increased by 100 mJ/cm^2^; if the erythema reaction time was 1 week or more, the original dose was maintained. After each treatment, cold compress and sun protection are necessary.

Data collected from the patients included gender, age, time to first onset, the anatomical location of the lesion, treatment duration, onset of repigmentation, and changes in the size of the skin lesions before and after treatment.

### 2.2 Assessment of Responses

The formula for calculating the recovery rate of vitiligo lesions involved the division of the difference between the lesion areas before and after treatment by the initial lesion area. Classification by repigmentation level: 0 ≤ ratio < 25%, 25% ≤ ratio < 50%, 50% ≤ ratio < 75%,75% ≤ ratio ≤ 100%. Repigmentation of < 25% was considered poor; repigmentation of ≥ 75% was considered acceptable, and repigmentation of ≥ 90% was considered excellent.

### 2.3 Statistical Analyses

Data processing and analysis were performed using R version 4.4.0, along with Zstats 1.0 (www.zstats.net).

## 3 Results

### 3.1 Patients' Demographic Data

104 patients were treated, including 38 males and 66 females. There were 181 skin lesions observed (110—head and neck, 55—trunk and limbs, and 16—acral). There were no statistical differences in the basic information of the above patients in the two groups (Table 1).

**TABLE 1:** Summary of patients' demographic data.

Patient characteristics	Group A	Group B	Statistic	*p*
Patients				
Sex, *n* (%)				
Male	15 (34.09)	23 (38.33)	*χ* ^2^ = 0.20	0.657
Female	29 (65.91)	37 (61.67)		
Age, mean ± SD (years)	33.20 ± 14.16	32.07 ± 15.68	*t* = −0.38	0.704
Skin lesions				
Location, *n* (%)				
Head and neck	44 (56.41)	66 (64.08)	*χ* ^2^ = 1.24	0.538
Trunk and limbs	27 (34.62)	28 (27.18)		
Acral	7 (8.97)	9 (8.74)		
Area, *M* (Q₁, Q₃) (cm^2^)				
Head and neck	2.25 (1.09, 5.44)	3.88 (1.80, 8.00)	*Z* = −1.73	0.084
Trunk and limbs	4.50 (1.88, 13.00)	3.00 (1.95, 9.25)	*Z* = −0.61	0.544
Acral	6.00 (3.00, 9.00)	3.50 (1.95, 4.00)	*Z* = −1.11	0.265

Abbreviations: *M*, Median; Q₁, 1st Quartile; Q₃, 3st Quartile; SD, standard deviation; *t*, *t*‐test; *Z*, Mann–Whitney test; *χ* ^2^, chi‐squared test.

### 3.2 Treatment Outcome

**FIGURE 1:**
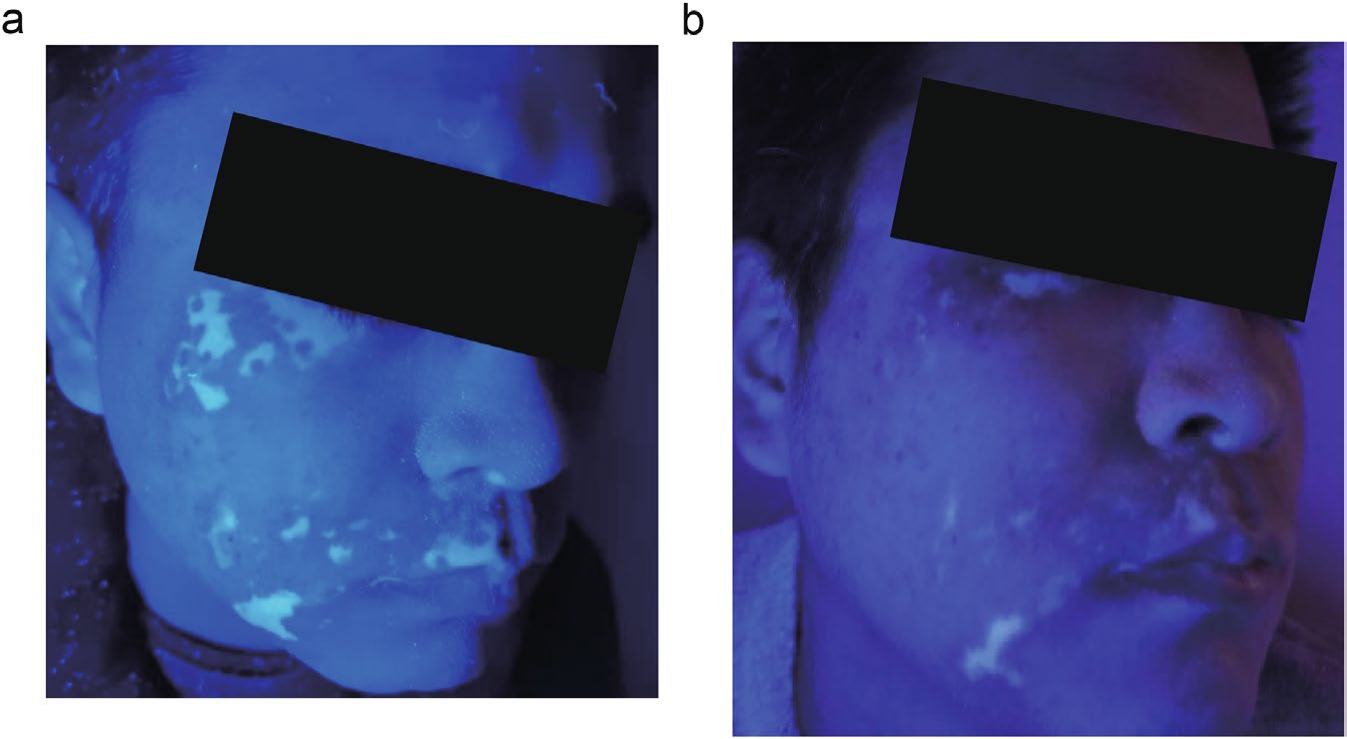
A patient receiving the 26‐week combination treatment with 308 nm excimer light and 2940 erbium laser. (a) Before the treatment, the patient's facial skin lesion condition. (b) 26 weeks after treatment, the patient's facial skin lesion status.

44 individuals in Group A, with 78 skin lesions, and 60 individuals in Group B, with 103 skin lesions, formed the basis of our research. The proportion of repigmentation responses in Group A with acceptable and excellent responses was significantly higher than that in Group B, and the proportion of poor responses in Group A was significantly lower than that in Group B (Table 2). Figure 1 shows a photograph of a patient with facial nonsegmental vitiligo before and after 26 weeks of combined treatment with 308 nm excimer light and 2940 erbium laser.

**TABLE 2:** Repigmentation assessment of Group A and Group B.

Repigmentation percentage *n* (%)	Group A	Group B	*χ* ^2^	*p*
[90, 100]	43 (55.13)	14 (13.59)	35.49	< 0.001*
[75, 100]	49 (62.82)	44 (42.72)	7.18	0.007*
[50, 75)	14 (17.95)	18 (17.48)	0.01	0.934
[25, 50)	11 (14.10)	20 (19.42)	0.88	0.347
(0, 25)	4 (5.13)	21 (20.39)	8.68	0.003*

*Note: χ* ^2^: chi‐squared test.

* *p* < 0.05.

### 3.3 Location and Efficacy

In Group A, there were 78 skin lesions (44 head and neck, 27 trunk and limbs, and 7 acral). Group B had 103 skin lesions (66 head and neck, 28 trunk and limbs, and 9 acral). Table 3 shows the repigmentation of skin lesions in different body parts in the two groups. There was a better response in different areas in Group A except for acral areas, where there was no significant difference.

**TABLE 3:** Repigmentation assessment of Group A and Group B based on the location of skin lesions.

Repigmentation percentage *n* (%)	Group A	Group B	Statistics	*p*
Head and neck				
[90, 100]	29 (65.91)	11 (16.67)	*χ* ^2^ = 27.66	< 0.001*
[75, 100]	32 (72.73)	31 (46.97)	*χ* ^2^ = 7.16	0.007*
[50, 75)	7 (15.91)	15 (22.73)	*χ* ^2^ = 0.77	0.381
[25, 50)	3 (6.82)	10 (15.15)	*χ* ^2^ = 1.76	0.185
(0, 25)	2 (4.55)	10 (15.15)	*χ* ^2^ = 2.06	0.151
Trunk and limbs				
[90, 100]	13 (48.15)	3 (10.71)	*χ* ^2^ = 9.34	0.002*
[75, 100]	16 (59.26)	9 (32.14)	*χ* ^2^ = 4.08	0.043*
[50, 75)	4 (14.81)	2 (7.14)	*χ* ^2^ = 0.23	0.631
[25, 50)	7 (25.93)	9 (32.14)	*χ* ^2^ = 0.26	0.612
(0, 25)	0 (0.00)	8 (28.57)	*χ* ^2^ = 6.88	0.009*
Acral				
[90, 100]	1 (14.29)	0 (0.00)	—	0.437
[75, 100]	1 (14.29)	4 (44.44)	—	0.308
[50, 75)	3 (42.86)	1 (11.11)	—	0.262
[25, 50)	1 (14.29)	1 (11.11)	—	1.000
(0, 25)	2 (28.57)	3 (33.33)	—	1.000

*Note: χ* ^2^, chi‐square test; —, Fisher exact.

* *p* < 0.05.

### 3.4 Analysis of the Factors Associated With the Efficacy

**FIGURE 2:**
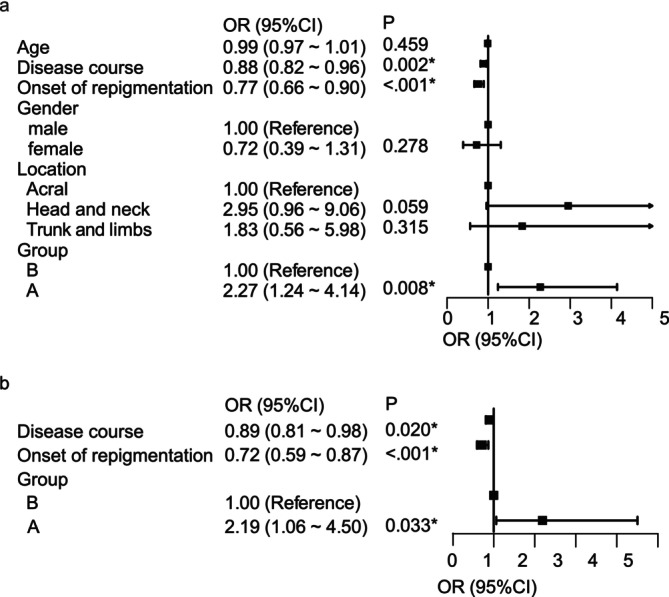
Univariate and multivariate analysis of the treatment efficacy. (a) Univariate analysis based on skin lesions with the repigmentation of 75%–100%. (b) Multivariate analysis based on skin lesions with the repigmentation of 75%–100%. * means *p* < 0.05 statistically significant.

Univariate analysis of the efficacy in the two groups was performed as follows (Figure 2a). Treatment group, disease course, and onset of repigmentation were the three factors that may affect whether the treatment efficacy can be acceptable. Choosing combination therapy, the shorter the disease course and the earlier the onset of repigmentation, the higher the likelihood of skin lesions in achieving acceptable outcomes. There was no statistical difference in the effect of age, sex, or the location of the skin lesions on achieving an acceptable treatment effect. Then, it was verified again in a multivariate analysis (Figure 2b). In multivariate analysis, we verified that choosing combination therapy, the shorter disease course, and the earlier onset of repigmentation were independent protective factors for achieving acceptable efficacy.

**FIGURE 3:**
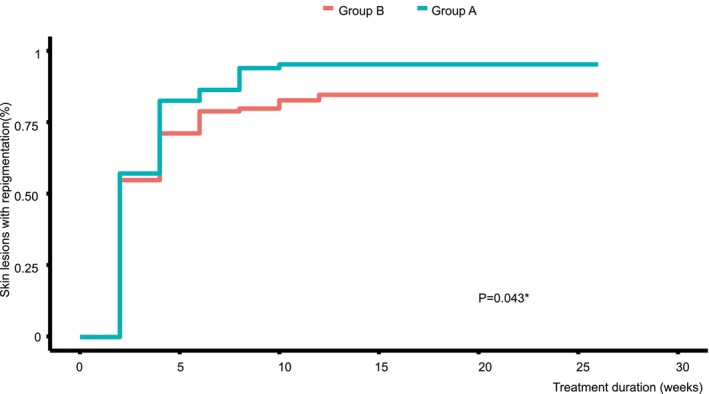
Kaplan–Meier analysis based on the treatment duration and onset of repigmentation in skin lesions.

Kaplan–Meier analysis of treatment duration and onset of repigmentation was performed as follows. Figure 3 shows the proportion of skin lesions with repigmentation in Group A and Group B at different treatment duration points. In the early stage of treatment duration, Group A had a higher proportion of skin lesions with repigmentation than Group B; after 26 weeks of treatment durations, the total proportion of skin lesions with repigmentation in Group A was more than that in Group B. It was 10 weeks the latest time of skin lesions to repigment in Group A and 12 weeks in Group B. In conclusion, in group A, the skin lesions responded earlier, and a higher percentage of skin lesions showed repigmentation within 26 weeks.

## 4 Discussion

Phototherapy, including psoralen‐UV‐A (PUVA) and NB‐UVB therapy, is the main treatment of vitiligo, while excimer light therapy and various topical drugs are used for the treatment of local skin lesions. However, phototherapy requires frequent outpatient visits and requires 6 months even longer treatment periods, sometimes producing unsatisfactory results. Therefore, the visit management of the patients with vitiligo is very challenging, and patient compliance and patient confidence in treatment are essential for successful phototherapy. A reference to the expected therapeutic response to phototherapy in vitiligo patients will aid in the management of the patients [28]. We hope that the improved efficacy of combination therapy can encourage the enthusiasm of patients for treatment and thus harvest better efficacy.

The 308 nm excimer light can accurately locate the area of skin lesions in vitiligo patients and accurately use a controlled dose of UVB radiation to decolorized patches, thus stimulating the activity of melanocytes and promoting the pigmentation of decolorized patches [29]. Research indicates that the treatment with a 308 nm excimer light on its own may provide advantages to individuals diagnosed with vitiligo. However, the outcomes frequently fall short of expectations, necessitating extended treatment durations to achieve desired results. The clinical efficacy of 308 nm excimer light is associated with the site of skin lesions and has a plateau after multiple treatments [30]. Combining 308 nm excimer light with other therapies has produced better results [15, 31]. The 308 nm excimer light combined with tacrolimus is more effective than treatment alone [32, 33]. Extensively researched is the effectiveness of combining 308 nm excimer light with the CO_2_ laser and UVB with the CO_2_ laser for treating vitiligo [34]. Numerous studies have shown that the combination of multiple treatments is more beneficial for the efficacy of vitiligo.

The 2940 erbium laser is a pulsed laser with a wavelength of 2940 nm, which is at the highest peak of water absorption and can accurately vaporize the superficial tissue of the skin. Compared to the CO_2_ laser, the 2940 erbium laser is highly selective for water, hence resulting in less potential thermal damage to the skin surface [35]. Its thermal effect contracts the tissue at the skin lesions and results in smaller skin lesions. At the same time, it can promote collagen remodeling and re‐epithelialization [36]. During the stage of reepithelialization and dermal regeneration, growth factors released can stimulate melanocyte division and proliferation within hair follicles [35]. Furthermore, the laser alters the molecular structure of the tissue, creating a compact pathway and enhancing the penetration of medications [22, 37, 38].

The 2940 erbium laser has high safety: permanent skin complications are rare; the immediate pain, erythema, edema and burning are mild and of shorter duration. Even in the high‐energy setting, it does not trigger the Koebner phenomenon. In practical clinical practice, this treatment option proves effective and safe for managing stable cases of vitiligo [39].

Our findings indicate a significant effect of combination therapy on vitiligo. Overall, skin lesions in Group A had better outcomes, except for acral. There is no difference in the proportion of poor efficacy in the head and neck, and we speculated that the head and neck itself may be sensitive to phototherapy, and most of the skin lesions will produce therapeutic responses. The poor response of vitiligo skin lesions in acral is not only because the site itself has been a clinical treatment problem but also related to its small sample size. Our cases came from patients, and relatively few patients had acral skin lesions; hence, there are fewer such samples.

Based on the above study and our outpatient treatment observation, most patients had rapid results within several months of 308 nm excimer light treatment, and then, some patients developed a plateau (skin lesions do not shrink or the narrowing rate slows down). Considering the efficacy, a combination treatment of 308 nm excimer light and 2940 erbium laser is recommended at the beginning for skin lesions in the head, neck, trunk, and limbs. For economic considerations, combination therapy can be administered after several months of 308 nm excimer light treatment to avoid the emergence of a plateau and thus improve the efficacy. At the same time, patients should adhere to the use of topical drugs, such as calcineurin inhibitors, hormones, etc., to improve the permeability of external drugs, and further promote color recovery. However, for patients with vitiligo at the hand and feet, our study showed that the efficacy of combination therapy was not significantly improved compared with the 308 nm excimer light alone. Therefore, surgical surgery may be a better choice for patients who failed 308 nm excimer light treatment, and new treatments for acral vitiligo need to be explored [40]. We found that during 26 weeks of treatment, all skin lesions that responded to treatment responded before 15 weeks, regardless of whether with monotherapy or with combination therapy. Thus, we can infer that most patients who respond to treatment will develop repigmentation within 3–4 months. If no repigmentation occurs at 3–4 months, the treatment should be stopped and other treatment options should be selected.

## 5 Conclusion

Combination therapy (308 nm excimer light combined with 2940 erbium laser) has a better efficacy on vitiligo than 308 nm excimer light alone. Combination therapy is a new option for patients when 308 nm excimer light treatment encounters bottlenecks.

## Author Contributions

All authors made substantial contributions to the conception and design of the work and the acquisition, analysis, and interpretation of data, drafted the work and revised it critically for important intellectual content, approved the version to be published, and agreed to be accountable for all aspects of the work in ensuring that questions related to the accuracy or integrity of any part of the work were appropriately investigated and resolved.

## Ethics Statement

The present study was conducted in accordance with the Declaration of Helsinki (initially published in 1964) on Ethical Principles for Medical Research Involving Human Subjects. Approval was obtained from the ethics committee of Huashan Hospital affiliated to Fudan University.

## Consent

Written or verbal informed consent was obtained from all patients prior to participation.

## Conflicts of Interest

The authors declare no conflicts of interest.

## Data Availability

The data that support the findings of this study are not openly available and are available from the corresponding author upon reasonable request.
